# Fish and complementary feeding practices for young children: Qualitative research findings from coastal Kenya

**DOI:** 10.1371/journal.pone.0265310

**Published:** 2022-03-14

**Authors:** Mary Kate Cartmill, Ivy Blackmore, Catherine Sarange, Ruth Mbeyu, Christopher Cheupe, Joaquim Cheupe, Elizabeth Kamau-Mbuthia, Lora Iannotti, Andrew Wamukota, Austin Humphries, Carolyn Lesorogol

**Affiliations:** 1 Brown School, Washington University in St. Louis, St. Louis, Missouri, United States of America; 2 Department of Human Nutrition, Egerton University, Nakuru, Kenya; 3 Department of Environmental Sciences, Pwani University, Kilifi, Kenya; 4 Department of Fisheries, Animal and Veterinary Sciences, University of Rhode Island, Kingston, Rhode Island, United States of America; 5 Graduate School of Oceanography, University of Rhode Island, Narragansett, Rhode Island, United States of America; Institut de recherche pour le developpement, FRANCE

## Abstract

This study examines barriers to fish consumption during the complementary feeding period in two coastal counties of Kenya with high rates of child malnutrition. Study findings indicate that young child fish consumption is impacted by factors related to accessibility, food preferences, and caregiver’s knowledge and beliefs about fish during the complementary feeding period. These factors are influenced by prominent community figures such as elder women and health workers, whose own beliefs and understandings are impacted by underlying cultural norms, potentially limiting fish consumption. To our knowledge, this is the first study conducted in the coastal region of Kenya to focus on understanding fish consumption attitudes and beliefs during the complementary feeding phase. Our findings represent a critical first step towards the creation of more effective policies and interventions to address the significant nutritional disparities that exist in the study population.

## Introduction

In Kenya, approximately 26% of children under five are stunted, notably higher than the global prevalence. On the Kenyan coast, stunting rates are almost double the national rate. In the coastal counties of Kilifi and Kwale, 52% and 40.2%, respectively, of children under 5 are stunted [1]. Research examining the reasons for the high prevalence in this region is limited. Existing literature points to poor dietary diversity during the complementary feeding phase as a potential contributor [2, 3]. In order to develop effective strategies and interventions to reduce stunting and improve health and development outcomes, more research is needed to understand the drivers of feeding practices during this critical growth phase.

Animal source foods (ASFs) provide important nutrients in highly bioavailable forms [4]. Consumption of these foods during pregnancy, the complementary feeding period, and in early childhood has been found to reduce rates of stunting in low-income populations [5–11]. Nutrient-dense ASFs are especially critical between six and twelve months when nutrient needs are high and energy intake from complementary foods is relatively low [12, 13]. Despite their nutritional benefits, rates of ASF consumption remain low in Kenya [1]. Fish are a unique source of ASF containing most of the minerals, vitamins, polyunsaturated fatty-acids (PUFAs) and micronutrients necessary for proper growth and development including iron, Vitamin A, calcium, iodine, zinc, selenium, DHA and Vitamin B-12 [14]. Fish increases absorption of plant-based micronutrients, particularly beneficial in populations where cereal-based staples comprise the majority of the diet [15–17]. Recent studies suggest that children who consume higher levels of fish are more likely to meet daily nutritional requirements and have better growth outcomes than those who do not [18, 19].

Over two million Kenyans rely on fishing and fishing-related activities for their livelihoods [20]. In 2019, earnings generated from marine fisheries exceeded 4.7 billion Kenya shillings (about 43 million USD) [21]. Previous research shows that fish-related livelihoods have the potential to improve household nutritional security [22–25], but participation in fishing activities does not guarantee higher consumption of fish foods in the household [26]. These findings indicate a more complex pathway exists between catching and consuming fish. For instance, there are about 13,000 fishing households in the coastal region of Kenya [27], yet high rates of child malnutrition remain a concerning and persistent problem.

Few studies have examined perceived barriers to fish consumption during the complementary feeding phase in coastal fishing communities. In Indonesia, Gibson et al. [18] found that beliefs in fish causing allergies or illness in young children delayed their introduction in childhood feeding. Research in Bangladesh found that fish was commonly withheld from young children’s diets until after 6 months of age due to fear of children choking on bones [28]. In Kilifi County, one of our study sites, Mbogoh, Nanua and Shauri [29] found that negative beliefs about the effects of fish foods restricted consumption during pregnancy and lactation but not in complementary feeding. High levels of endorsement of these beliefs, however, was associated with elevated rates of child underweight and wasting. Distribution of ASFs within the household has also been shown to negatively impact child intake in many low- and middle-income settings [30, 31].

Additional studies in Kenya highlight potential barriers to overall household fish intake ranging from economic factors to household gender dynamics. Cornelsen et al. [32] identified price as a primary barrier to household fish consumption in two low-income areas of Nairobi. Esilaba, Moturi and Mokua [33] also identified price as a primary barrier to higher consumption among customers at a major fish market in Nakuru town. The export market for Nile Perch from Lake Victoria was found to limit fish access and negatively impact food security in countries bordering the lake [34–36]. Fiorella et al. [26] suggest that gender may play a role in whether a fishing household consumes fish as men often decide whether to sell or save fish for the family to eat.

This article presents results of a qualitative investigation conducted as part of a mixed-methods formative research study carried out in 2019 in two coastal counties (Kilifi and Kwale) in Kenya. The study aimed to better understand current complementary feeding practices, how households in the study communities perceive the nutritional value of fish, and potential barriers and facilitators to feeding fish during the complementary feeding stage. Understanding the factors influencing fish consumption represents a critical first step towards creating better policies and interventions to address the significant nutritional disparities that exist in the study population.

## Materials and methods

### Study design

To pursue the aims noted above, this qualitative study included key informant interviews with nutritionists and community health workers (CHWs), and in-depth interviews and free list exercises with primary caregivers of children between 0 and 6 years. Study team members conducted twenty interviews, twelve with caregivers and eight with nutritionists and CHWs. Key informants were purposively chosen based on their knowledge of child nutrition and their role as health educators in study communities. Caregivers were selected based on their proximity to the study locations.

Interviews were conducted using semi-structured survey instruments developed by study team members (S1 and S2 Appendices). The questionnaires were developed in English and translated to Kiswahili by the research staff. Interview questions for caregivers focused on current family and child fish consumption; attitudes, behaviors, and beliefs around complementary feeding; perceived access to fish foods; and knowledge of how nutrition contributes to child growth and development. Caregivers were also asked to list food items that are considered part of a healthy diet for children. Free listing provides insight into the cultural domain of healthy diets for children and indicates the importance attached to different food items. Food items listed first or more frequently generally reflect higher salience to the study community [37]. Nutritionists and CHWs were asked about their roles and activities as healthcare providers; perceptions of fish consumption in the community; understandings of child growth and development; and promotion of fish in child diets.

Interviews were conducted in the participant’s home or other private location after obtaining informed consent. Interviews took on average 30 to 45 minutes and were recorded using a digital voice recorder. One study team member conducted interviews while another team member recorded detailed notes. The interviews were conducted, recorded, and transcribed in the local language of Kiswahili. The transcripts were then translated into English for analysis by the research team.

Ethical approval for this study was obtained from the Human Resource Protection Office of Washington University in St. Louis, the Pwani University Ethics Review Committee and the Office of Research Compliance at Mississippi State University. Written informed consent was obtained from all participants and participation was on a voluntary basis.

### Study sites

The study was carried out in four communities in the coastal region of Kenya: Uyombo and Vipingo in Kilifi County and Tiwi and Shimoni in Kwale County. Study sites were chosen based on established relationships with the research team as well as their proximity and access to marine resources. Kilifi county covers an area of 12,370 km^2^ with a population of 1.45 million and average household size of 4.8 persons [38, 39]. The poverty rate is 46.4% and child stunting is 52% [1, 40]. It has five Agro-Ecological Zones (AEZ) suitable for different agricultural and livestock uses ranging from ranching to farming activities like tree-cropping and food-crop production [38]. The majority of the coastal region’s population identify as Mijikenda, a composite group including nine different ethnic groups. In Kilifi, the most populous ethnicity is Giriama and most practiced religion is Christianity.

Kwale County is located about 40 km south of Mombasa and covers an area of 8,267 km^2^, with a total population just under 867,000 and an average household size of 5 [39, 41]. Kwale also has five AEZs. The most prominent ethnicity in Kwale is Digo and the most common religion is Islam [40]. Compared to Kilfi, Kwale has lower rates of stunting in children under five years old (46.4%) [1] but a slightly higher poverty rate of 47.4% [40].

Fishing is an important economic activity in Kilifi and Kwale Counties and, although the caregivers interviewed for the qualitative portion of the study were mostly not directly engaged in fishing activities, they do live in close proximity to those who are and therefore are expected to have relatively easy access to fish.

### Data analysis

The qualitative data analysis was performed by the lead author in collaboration with the co-authors. Interview transcripts were uploaded into Nvivo 12 for Mac. Cases were created for each interview and classified as either a caregiver or key informant. Transcripts were read and coded to identify key information relevant to the study questions. A coding structure consisting of primary and sub-codes was developed deductively, based on the areas of focus in the interview instrument, and inductively as new information emerged [42]. Examples of primary and sub-codes are shown in Table 1. Primary codes clustered around perceptions and practices related to fish and seafood consumption for young children and families, barriers and facilitators of consumption, and knowledge of connections between fish consumption, nutrition, health and child development. Fish in this context is defined as ray-finned fishes (Class Actinopterygii), including elasmobranchs, and seafood is defined as including other aquatic species like crustaceans, and mollusks, including octopus, but not including aquatic plants.

**Table 1:** Example of primary and sub-codes.

Primary Code	Child and family feeding practices and beliefs	Fish and seafood consumption—perceptions, knowledge, barriers	Maternal and child health knowledge
**Sub-code 1**	Child food consumption of fish and seafood	Barriers to fish and seafood consumption	Food, nutrition and feeding promotion
**Sub-code 2**	Early childhood feeding practices and beliefs	Fish cost	Child growth and development knowledge sources
**Sub-code 3**	Barriers to consuming Animal Source Foods	Perceptions and beliefs about fish and seafood consumption	Nutrition, health and child development

**Fig 1:**
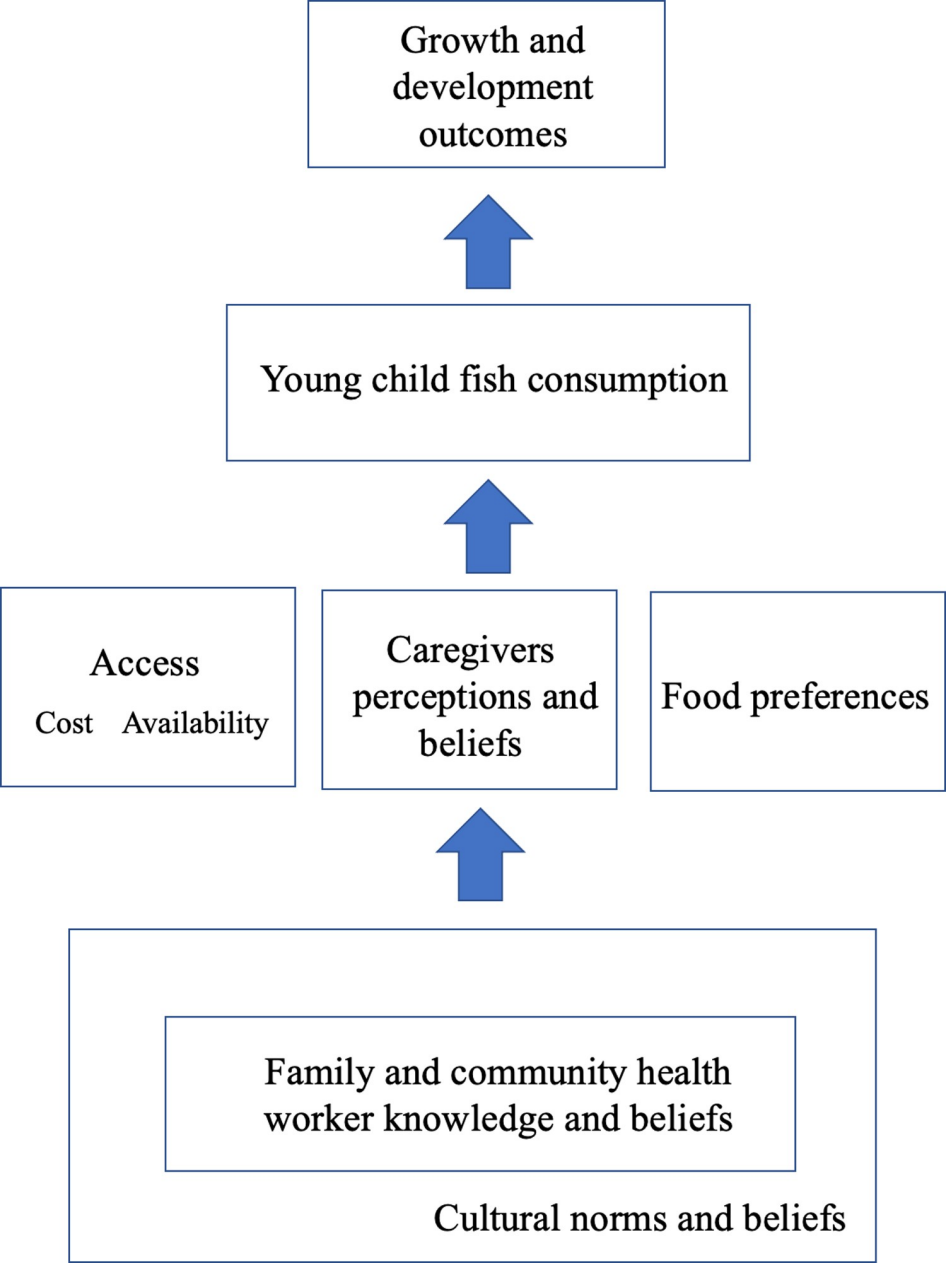
Conceptual framework of factors influencing child fish consumption during the complementary feeding period in Coastal Kenya based on study findings.

Further analysis of the codes resulted in identification of the major factors and pathways influencing decisions to feed fish in the complementary feeding period, which are illustrated in the conceptual framework shown in Fig 1.

The most immediate factors influencing feeding fish as complementary food (in the center of the figure) are access to fish foods, caregiver perceptions and beliefs, and food preferences. The nature of influence varies depending on geographic and social context. For example, caregivers may hold positive beliefs about the nutritional value of fish for child growth and development but have limited access to fish due to high cost or low availability. As a result, they may prioritize lower cost, less nutritious foods during the complementary feeding period. Conversely, fish foods may be readily available in an area due to proximity to the ocean, accessible markets or intermediate sellers, but individual preferences for other foods or negative experiences with fish may limit consumption by the individual or in the household.

These proximal factors—especially caregiver’s beliefs and knowledge—were reported to be impacted by the knowledge and beliefs of influential family members, community members and CHWs who provide information on infant and young child feeding practices. These individuals can influence a caregiver’s beliefs about fish consumption in a variety of ways, depending on their level of influence and personal understandings of nutrition and child growth and development. CHWs also influence caregivers’ knowledge and beliefs through community education activities or clinic visits. When in agreement, these influential figures may reinforce positive messaging and drive fish consumption. Discordant views may lead to more uncertainty regarding the advisability of feeding fish to young children. Finally, the knowledge and beliefs of influential figures are shaped by prevailing community beliefs, traditions and practices around fish consumption. In the results and discussion below, these factors are explained more fully and supported with evidence from the interviews.

## Results and discussion

### Demographic characteristics

Demographic characteristics of the research participants are summarized in Table 2. All caregivers interviewed were female mothers or grandmothers between the ages of 18 and 72 currently caring for at least one child between the age of 0 and 6 years old. Three of the health workers interviewed were nutritionists and five worked as CHWs.

**Table 2:** Interview participant demographic characteristics.

Demographics		Caregivers	Health Workers
**Location**			
	**Kilifi County**	6	4
	Uyombo	3	2
	Vipingo	3	2
			
	**Kwale County**	6	3
	Tiwi	3	2
	Shimoni	3	2
**Occupation / Household Status**			
	Community Health Worker	N/A	5
	Nutritionist	N/A	3
	Fish Trader	2	N/A
	Non-Fish Trader	10	N/A
	Total	12	8

### Introduction of complementary foods

The World Health Organization (WHO) recommends exclusive breastfeeding for the first six months of life, after which children should continue to breastfeed while consuming a minimum of two meals per day between 6–8 months and 3+ meals per day between 9–23 months. Children older than six months are encouraged to eat meat, poultry, fish or eggs daily and to consume foods from at least four of the seven food groups to reach the recommended minimum dietary diversity [43]. The Kenyan food-based dietary guideline differs somewhat from the WHO guidelines, recommending the introduction of staple porridge at six months and more diverse foods starting from 7–8 months. At 12 months of age, breastfed children are encouraged to consume 125 ml of family foods with one food from each food group (ASF, staple, legumes and seeds, and fruit and vegetables), doubling the amount by 23 months while maintaining a minimum dietary diversity of one food from each food group [44].

### Fish consumption in complementary feeding in study communities

The introduction of fish during the complementary feeding period was generally low compared to other foods. Most caregivers reported feeding porridge made from maize, millet or cassava flour as first foods. Reported age at which caregivers introduced fish to their children is summarized in Table 3. Half of caregivers began including fish when the child reached one year old, and those who introduced fish between six and eleven months mostly served it in the form of a soup or broth, not including the meat, organs or bones of the fish. This practice likely reduces the full benefits of feeding fish as many of the important nutrients are located in the meat, bones and organs [5, 45].

**Table 3:** Number of respondents who introduced fish to their child’s diet.

Age (in months)	Fish Soup	Fish Meat
0–6 months	1 (8.3%)	0
6–12 months	4 (33.3%)	1 (8.3%)
12+ months	5 (41.7%)	1 (8.3%)

Reported frequency of feeding fish to children was also below the recommended levels [43]. Just over half of caregivers interviewed reported feeding their children fish on a weekly or regular basis. Of those, two reported feeding their children fish two to three times per week and one, a fish trader, reported feeding fish to her children on a daily basis.

### Access to fish and seafood

#### Cost

Fish access appears to play a major role in whether or not caregivers feed fish to their children and families. As illustrated in the conceptual framework (Fig 1), cost and availability are the two primary factors determining access to fish. Over half of respondents identified the cost of fish as a barrier to feeding fish to children, making it the most common access-related barrier. In some cases, the high cost of fish as compared to other food items outweighed positive associations and preferences, as explained by one CHW:

…*perhaps the economy is the only thing that can determine*. *Maybe one doesn’t have the money to purchase fish, despite them liking the fish and desiring it, they are unable to buy and feed their children, unless one is from a fishing household*. *(Interview 04)*

This response includes an assumption that fishing households have greater access to fish, but another caregiver engaged in fish trading explained how this might not always be the case. Even though she reported frequent consumption of fish in her household, she also identified financial barriers to providing fish for her children, indicating that the cost of fish can outweigh preferences for and access to fish.

Both caregivers and health workers explained that the cost of fish varies depending on fish quantity and quality as well as the purchase location. In Kilifi, most respondents reported that fish were less expensive if purchased directly from the shore than in shops or markets. The additional costs of transport and freezer storage were identified as reasons for the higher cost in shops. Those who cannot directly access the shore end up paying a higher price to consume fish. As one caregiver from the Vipingo community explained:

*If you get fish from the sea shore it is very cheap, although I have never gone there to get fish*. *At the fish shop they increase the price because of the cost of getting fish from the seashore and storage in the freezers. (Interview 17)*.

In Kwale, responses were mixed, with similar numbers of respondents reporting higher prices at markets compared to the shore and at the shore compared to markets or shops. This variance reflects variables related to the value chain for fish. In Tiwi, for example, one CHW described how fish can be purchased directly from fishermen, at local shops or from *mama karanga* (i.e., the traditional name for women who purchase fish to fry and sell to consumers). In her description, proximity to fishermen increased access to fresh fish, whereas distance from the shore reduced access and increased reliance on intermediaries like shops or *mama karanga*:

*Not all fish comes from the shore. Those who live close by the fishermen are the ones that get fish from the shore*. *Then there are a few fishermen who leave some behind for local sale and that’s how we sometimes get the fresh fish from the shore….The Una is brought from Likoni or Mombasa*, *someone goes for them, then either sells fresh or deep fried. Women may purchase the deep fried or fresh while others purchase the fresh and go to deep fry and sell them, too. (Interview 04).*

Another factor limiting access to fish is the gendered nature of fish markets. Participants explained that women primarily purchase fish from shops, *mama karanga* or fishermen selling in communities, whereas men and *mama karanga* purchase fish directly from the shore. Thus, women’s access to fish is limited by the market outlets that they have access to. The following response indicates women’s reliance on men for access to fish:

*I normally buy fish from the fish shop, but there are weekends when my husband has not gone to work and he accompanies the fishers around here to the beach where he also does fishing, but it is just for our family*. *In case he does not do the fishing then he buys fish from the fishermen at the sea shore (Interview 16)*.

This also suggests that access to less expensive fish at the shore is restricted to men and women traders engaged in the fish value chain.

#### Availability

A few interviewees from both counties reported varying availability of fish affecting their ability to purchase it throughout the year. Inclement weather and seasonal weather patterns that affect fishing were cited as impacting the abundance of fish. Previous research confirms the seasonable availability of fish in Coastal Kenya, with higher quantities caught during the Northeast monsoon season (October to March) than during the windier Southeast monsoon season (April to September) [46]. Variation in availability causes price fluctuations [47]. When fish are more plentiful, prices drop, enabling higher fish consumption in the community, as described by one CHW:

*There are seasons when we have plenty [of fish] and some when we don’t. During high seasons you can get fish at twenty shillings*. *They contribute to proteins because we eat a lot of it. (Interview 09)*

Conversely, some respondents reported purchasing less desirable types of fish when prices are high or more desirable varieties are unavailable, revealing how both price and availability influence purchasing decisions. In Vipingo, several caregivers reported substituting tuna for more desirable local fish when it was not available or too expensive. As one caregiver explained:

*We normally get fresh fish from the sea, but at the moment fish is not easily available due to the weather so I [buy] tuna from the fish shop*, *but when fish is available I buy the other types, because tuna is not good, since it is fed artificial foods for it to attain a certain weight.(Interview 15)*

Seafood, especially crabs, prawns and oysters, while not as familiar to some caregivers and health workers, was reported to be expensive and hard to access. Caregivers from Kilifi County discussed how demand from the local tourist market drives up the price and limits the availability of seafood:

*My children like oysters and crabs, but they are expensive and in high demand in the hotels around*, *so their availability is also a challenge. (Interview 12)*

Informal networks of fish exchange or gifting in the community may also increase access to less marketable seafood items, as suggested by this caregiver:

*The big crabs are sold to the hotels*, *but small crabs do not have a market so the fisherman normally boils them and shares them out to us, since we are neighbors. (Interview 15)*

Overall, our results suggest that access to fish is constrained by high prices, gendered purchasing patterns and fluctuating availability. Limits on access help explain the low levels of consumption, especially in poorer communities with limited ability to increase spending on food.

### Caregiver beliefs, knowledge, and perceptions about fish

#### Importance of fish in the diet

Beliefs regarding the nutritional value of fish varied across the participants. When asked to free list foods that should be included in a healthy diet for young children, half of caregivers included fish, but none listed it as the first item (Table 4). Most of the foods listed higher are starchy staples or grains such as maize, maize porridge, plantains, potatoes and rice. In free listing, the order and frequency in which responses are given reflect the personal or cultural importance of the items listed [37]. Based on this understanding, starchy staples were considered a very important component of a healthy diet by respondents and fish was considered less important. This finding is consistent with dietary data from the quantitative component of the study, which found that maize and starchy foods make up the majority of children’s diets.

**Table 4:** Free list of foods identified as part of a healthy diet for young children.

Kwale County					
Shimoni	Shimoni	Shimoni	Tiwi	Tiwi	Tiwi
Maize	Potatoes w/ coconut milk	Bananas	Maize Porridge	Maize	Amaranthus
Beans	Rice and stew	Porridge	Beans	Maize Porridge	Cabbage
Corn meal			Rice	Beans	Kale
Rice			**Fish Soup**	Rice	Beans
**Fish**			Meat	Greens	Rice
Vegetables				**Fish**	Chapatti (flat bread)
				Eggplant	Meat
				Amaranthus	Cornmeal
				Kale	Mangos
				Cabbage	Bananas
					Oranges
**Kilifi County**					
**Vipingo**	**Vipingo**	**Vipingo**	**Uyombo**	**Uyombo**	**Uyombo**
Plantains	Milk	Samosa	Sweet Potatoes	Beef	Rice
Ripe Bananas	Vegetables	Potatoes	Irish Potatoes	**Fish**	Beans
Potatoes	Amaranthus	Maize Porridge	Millet porridge	Vegetables	**Fish**
Maize Porridge	Beef	Rice	Beans	Beans	Irish potatoes
Soup	Potatoes	Beans	Cow’s milk	Milk	
Oranges	Chapatti (Flat bread)	Green Grams			
	Maize Porridge	**Fish**			
	Rice	Beef			
	Spaghetti	Flatbread			

In another free list exercise, caregivers ranked protein-rich foods in terms of frequency and importance in the diet. Almost all respondents from Kilifi County ranked fish as the most important protein, but only one respondent from Kwale County did. One reason for this difference may be the higher importance given to foods that can be grown at home. As one CHW from Shimoni in Kwale County explained:

*Fish comes in at position three because it is purchased*. *The top position is given to eggs followed by foods that are grown at the farm like beans, green grams [mung beans] and cow peas and eggs. (Interview 09).*

This sentiment was also reflected in the beliefs of a caregiver from the same county who described healthy foods as ones that she can grow herself:

*What I have the ability to get, such as the maize, we have it in our farms. We just mill the grains and cook Sima [maize porridge]*, *or we cook maize mixed with beans. Sometimes we cook together with rice or greens or even fish when we have purchased it, or brinjals [eggplant]. These are the foods that we mostly consume, because they are available in our farms. Others also include amaranthus, kales, cabbage. (Interview 02)*.

These statements reinforce the role of accessibility in household food consumption and its relationship to the perceived importance of foods. Foods that are more readily accessible tend to be highly valued, suggesting that even if fish is considered to have nutritional value, lack of availability may undercut that value in practice.

#### Positive perceptions and beliefs

The majority of caregivers expressed positive perceptions of the nutritional contribution of fish to children’s growth and development when asked directly, citing reasons like it being *‘energy giving’ (Interview 05)*, having *‘important minerals for the body’ (Interview 11)* and its ability to ‘*build the body’ (Interview 15)*. Some elderly caregivers expressed a cultural belief that consuming specific parts of the fish can benefit different aspects of child development, such as the fats in the fish head improving child brain development:

*Interviewer*: *Do you give your 2-year-old grandchild fish?*

*Respondent: Yes, we do feed him with fish because we know he requires it for his growth and development, so normally [we] remove the bone and give [him the fish]*. *Fish is a protein source food and the fish head is also good for children because it has good fats for brain development. (Interview 12).*

Other benefits of consuming fish identified by caregivers extended to the health and well-being of the mother, primarily in the form of increased milk production, in addition to contributing to child growth and development:

….*we also take needle fish, pono, shark, nguru, octopus and squid, although we prefer octopus to squid, because the octopus is known to enhance breast milk production in lactating women…Yeah the children grow well when they are fed with fish and they also become more intelligent*. *In fact, I like giving my grandchild the fish brain, because I believe that it improves the child’s intelligence. According to my perspective fish is good compared to beef and other meats*. *I have heard that consuming a lot of beef leads to arthritis and high blood pressure, but I have never heard any side effects of consuming fish apart from the octopus that leads to allergy. (Interview 17)*

Here the caregiver expresses a preference for fish over other animal source foods that she associates with chronic health conditions such as arthritis and high blood pressure. She echoes the idea that consuming the fish head improves child intelligence. This suggests a possible wider cultural belief regarding fish heads and intelligence, which will be explored further below.

#### Negative perceptions and beliefs

Allergic or negative physical reactions to consuming some species of fish and seafood were cited as common barriers to consumption, second only to cost. Reported reactions in children and adults varied from rashes to swelling, nausea and mouth sores. During one interview, a caregiver was asked if there were reasons not to feed children fish and identified Una [Indian Mackerel] as a potential source of negative reactions:

*My eldest child complains that he belches when he consumes Una*, *his stomach aches and he stays the whole day not happy thus he doesn’t like the Una fish. (Interview 02)*

The same caregiver also cited a negative reaction (swelling) to Una as the reason why she does not it eat herself. Other fish identified as potential sources of illness included papa, tafi, octopus, and tuna. Despite these physical reactions, price appears to outweigh potential health implications. As one caregiver noted:

*Consuming Una when your body temperatures are high results in mouth sores*. *However, we overlook this side effect due to financial constraints. (Interview 03)*

Several women reported bones as a reason to avoid feeding fish to young children. Caregivers identified certain fish that got stuck between children’s teeth (Eel) or had too many bones for children to eat. In one interview, a mother described how a fear of bones influenced the way she prepared fish for her child at a young age:

*Interviewer*: *What made you not feed your child the fish?*

*Respondent*: *I used to just fear because of the bones, so I would boil and give the soup or stew and give her the stew. (Interview 15)*.

In this case, the mother’s belief about the danger of bones directly affected the amount of fish her child consumed and the timing of introduction, as she reported feeding fish in this manner until after the child’s first year.

### Food preferences

Food preferences are another factor influencing the amount of fish in the diet and whether or not fish is introduced in complementary feeding. In addition to beliefs and perceptions about the nutritional value of fish, caregivers’ preferences for other foods and/or their dislike of fish are reasons for not including it in the complementary feeding period. In some instances it was the mother’s own dislike of fish that delayed its introduction, as described by a young mother from Shimoni:

*Interviewer*: *What about yourself, why did it take long before you introduced your child to fish yet you are closer to the ocean?*

*Respondent*: *It is because I don’t like fish. I prefer beans. (Interview 05)*.

This same mother reported fish as the least important protein in her household and did not introduce it to her child until nine months of age. In other cases, caregivers reported that their children disliked certain types of fish or seafood, which lowered their probability of cooking it for the family:

*Respondent*: *Yes I have cooked the octopus but rarely, not very common. However, my youngest child doesn’t like the octopus*.

*Interviewer*: *Is it that the octopus has a negative effect on him or he just doesn’t like it? Respondent: He just doesn’t like it. (Interview 02)*

Together these comments indicate that food preferences influence food choices and consumption in the household. If caregivers or children dislike eating fish, it is less likely to be included as a complementary food.

### Community influence on complementary feeding

Caregivers’ decisions about feeding fish in the complementary feeding period are also influenced by other members of the community. Most respondents reported receiving information about child feeding, growth and development from a mix of sources including family members such as mothers, mothers-in-law or siblings. Nankumbi and Muliira [48] used the term ‘influence of cultural custodians’ to describe a barrier to proper infant and young child feeding generated from cultural practices passed to mothers from respected family or community members. A similar pattern of generational and community influences emerged from the interviews with caregivers and CHWs. Influences included ideas about which types of fish to feed to young children, the duration of exclusive breastfeeding and timing of initiation of complementary feeding. In many cases, elders’ advice conflicts with the official recommendations of the Ministry of Health, putting healthcare workers in the difficult position of recognizing the respected role of elders in society while also encouraging women to follow the science-based recommendations.

Elders seem to play a particularly influential role in complementary feeding practices and beliefs, as evidenced in the following interaction with a middle-aged Vipingo caregiver:

*Interviewer*: *Okay, have you heard that fish should not be given to children?*

*Respondent*: *Yes. There is a specific type that is not given to children, but I cannot remember the name of that fish*.

*Interviewer*: *Why should that specific type not be given to children?*

*Respondent*: *I really do not know the reason, but we are just told by the elders not to give children that type of fish. (Interview 16)*

Elders’ influence on child feeding decisions can take the form of actions as well as advice, especially since elder women (mothers, mothers-in-law) often share caregiving roles with mothers. For example, earlier in this same interview, the mother described how the grandmother’s decision to give the baby water led to the initiation of complementary feeding:

*Interviewer*: *What foods did you start giving her at 5 months?*

*Respondent*: *It was her grandmother that gave her water. (Interview 16)*

Some evidence also emerged of dissonance between the information provided by maternal figures and the practices promoted by healthcare workers, as shown in these comments from health workers:

*Interviewer*: *Do mothers also seek advice from their mothers- in- law?*

*Respondent*: *Yes, we always discourage them from seeking their advice, because they always say what is contrary to what we advise them. So we tell them to listen to them but follow what we advise them. (Interview 13)*

*Most of them regarding matters of exclusive breastfeeding, there are misconceptions especially from family members, for example from their grandmothers. So mothers tell you the opposite of what you know*. *And since it’s their mothers- in- law [that] has told them, they have to do it. If you ask them at the clinic they inform us ‘so-and-so’ told them. Then we correct them. (Interview 20)*

*Those who have mothers-in*- *law consult them, but usually they discourage them from exclusive breastfeeding claiming that the child remains hungry (Interview 09)*.

In each case, the health care worker identified differences between what women are advised by their elders and the recommendations provided by health care workers. Recognizing the respect given to elders in the community, the healthcare workers did not advise women to disregard what they were told by their elders, but rather to follow the professional recommendations in practice.

#### Healthcare worker influence

In addition to family members, hospitals, clinics, and CHWs were identified as primary sources of information on child nutrition, growth and development. All healthcare workers interviewed reported promoting fish for child feeding as part of their community education practices. Most health workers correctly identified nutritional benefits in fish such as minerals, vitamins, proteins or omega 3 and 6 fatty acids. One theme that emerged from the interviews with healthcare workers was a common belief in the nutritional superiority of boiling fish over frying as a preparation method. Contrary to this belief, nutrition literature suggests that the process of frying fish is unlikely to destroy its nutritional value and may even enhance absorption of certain nutrients, including fat soluble vitamins like A, D, E, and K [45, 49].

One healthcare worker explained how she promotes fish and the idea that frying fish destroys nutrients:

*Yes I always encourage them to take fish*, *because fish provides the body with good nutrients, and I encourage them to take boiled fish in order to get all the nutrients from fish because when you deep fry fish you destroy all the nutrients. (Interview 14)*

Another described her belief that frying interferes with the nutritional value:

*Most people around here are fishermen. I educate them to boil fish and add some salt, but they prefer to deep fry with plenty of pilpili [pepper]*, *mango, tomatoes, onions and tangawizi [ginger], thus interfering with the nutritive value of fish. (Interview 08)*.

Only one nutritionist reported no nutritional differences between the preparation methods but still promotes boiling over frying as a way to make it easier for younger children to eat fish.

Evidence of the health workers’ instruction and messaging is reflected in the statements of caregivers who claimed that boiling fish is a healthier preparation method. Some interviews suggest that messaging around boiling fish may be misconstrued by caregivers to mean that the broth of boiled fish is equally nutritious as the meat, potentially contributing to the practice of feeding fish soup (without meat) to children. Healthcare workers confirmed witnessing this practice, despite their attempts to dispel the idea:

*Interviewer*: *Are children fed on fish itself or only the soup?*

*Respondent*: *Most children are fed only on soup while some are given the flesh too*.

*Interviewer*: *So, they believe that giving them soup is similar to giving them flesh. Have you educated them about this misconception?*

*Respondent*: *I always educate them but practicing is the challenge. (Interview 08)*

Despite most health workers’ understanding of the importance of feeding fish flesh in complementary feeding, few mothers expressed knowledge of the difference between feeding fish flesh or soup alone. One mother who reported learning about healthy foods and child feeding from nutritionists and CHWs exhibited the belief that soup alone was healthy:

*Fish is good for children*, *especially when you boil fish and give your child the soup*. *It gives them good vitamins and makes them healthy*. (Interview 16).

Although underlying drivers of feeding broth or soup to children likely include barriers such as the high cost of fish or fear of bones, messaging from nutritionists and CHWs may be inadvertently bolstering the idea that fish soup alone is nutritionally sufficient. This dynamic highlights the diverse influences of community figures. Findings of familial or community influence on child feeding practices have been confirmed in previous studies in both Kilifi County and Kwale County [50–52].

### Broader cultural norms and beliefs

The remaining factor in the conceptual framework is the influence of cultural beliefs and norms regarding feeding fish. Interviews revealed a number of beliefs, some of them contradictory, about feeding fish to children. As noted above, some caregivers believe that feeding the fish head improves child intelligence. Others reported that fish could be dangerous for children. For example, one CHW explained how the tradition of avoiding fish heads leads to lower consumption:

…*they say that children should not eat a lot of fish, or if they consume fish they should not eat the fish head*. *Because it’s a tradition they have that if the child eats the head they will not be able to understand anything in school. So, they reduce what was to be given to the children because of culture*. *(Interview 04)*.

The advice she described giving them in return was:

…*leave the traditions*. *The head is also fish. So give the children fish to eat. First of all the fish comprises of the head and the tail*, *too, so children should consume the whole fish. (Interview 04*)

In another example, cultural norms regarding intra-household food distribution arose in one interview that indicated how these practices could restrict children’s fish consumption. The following interaction between a nutritionist and the interviewer highlights how adults are prioritized in food distribution within the household:

*Respondent*: *I had the opportunity to go to the community and what they do is buy 3 pieces of fish, prepare a very big ugali [maize meal], and the entire family will have to feed on that*. *The fish is served to the adults and maybe one piece shared among the rest of the children*.

*Interviewer*: *We thought that they probably think that children cannot take fish because of the bones*.

*Respondent*: *It is just culture, and most community members prioritize adults in food service. (Interview 13)*.

These examples show that broader beliefs and traditions in the community may restrict children’s access to fish. They also point to tensions between community beliefs and practices and the government’s current nutritional recommendations.

Results presented above demonstrate the variety of factors influencing complementary feeding decisions and the role of fish in complementary diets. A limitation of the study is the relatively small number of interviews that are not representative of all individuals involved in the complementary feeding process, including men. However, by interviewing primary caregivers, nutritionists and CHWs we were able to examine the current perceptions and uses of fish in complementary feeding, their promotion by healthcare workers and understandings of the connection between fish and child growth and development.

## Conclusions and implications

Fish and other aquatic foods are increasingly being recognized as playing a critical role in achieving food and nutrition security globally [53, 54]. Unfortunately, consumption of these foods during critical growth periods remains low, even in populations where many individuals rely on fishing for their livelihoods [1, 18, 26]. This study sought to better understand the fish consumption gap and the role of fish and fish production in young child health and nutrition in coastal Kenya. Findings from the qualitative component of the study suggest that young child fish consumption is impacted by a combination of factors related to accessibility, food preferences and caregiver’s knowledge and beliefs about fish during the complementary feeding period. Feeding choices are further influenced by advice from prominent community figures, such as elder women and health workers, and cultural norms and practices. The conceptual framework (Fig 1) illustrates the interacting factors ultimately affecting child growth and development outcomes, particularly stunting, which is much higher in the study population compared to national and global prevalence rates [1].

Current complementary feeding practices in the study communities show a low level of fish consumption in young children through delayed introduction, feeding small amounts of fish or only the broth of fish soup. Our results suggest that fish access is primarily driven by cost, proximity to locations where fish are sold, gendered purchasing behavior and the seasonal availability of fish foods. These findings are in agreement with previous studies that also identified cost and gender dynamics as barriers to consuming fish and other animal sourced foods in Kenyan households [26, 32, 33]. Caregivers’ knowledge, beliefs, and food preferences also play a role in decisions to feed fish to young children and overall consumption levels. In addition to reports of allergic or negative physical reactions to certain types of fish and a fear of choking on bones, many caregivers did not think of fish as being a key part of a healthy diet for their young child and had mixed perceptions regarding its importance as a protein source compared to other animal source foods and beans. A lack of understanding regarding the nutritional importance of fish in addition to the accessibility challenges may be reducing overall consumption, especially when cheaper foods are perceived to have nutritional value similar to fish.

Other factors influencing child fish consumption are the knowledge and beliefs of family members, especially grandmothers, health workers and broader cultural norms. Many caregivers indicated that they received information about child feeding, growth and development from their mothers, mothers-in-law or siblings. Though likely well-intentioned, much of this information was not consistent with WHO complementary feeding and Kenyan food-based dietary guidelines. Similar findings of familial influence on infant and young child feeding practices have been found in previous studies in the Kenyan coastal region and other LMICs [48, 50–52, 55]. The nutritionists and CHWs interviewed noted the challenges of countering cultural beliefs about when to begin feeding fish to children and/or the parts of the fish that children should be allowed to eat. However, they also impart the message that boiled fish is better than other cooking methods. Boiling fish often means children are fed only broth or soup, not fish flesh, which likely reduces the level of nutrients consumed and absorbed.

Findings from this study provide important insights that can be used to develop strategies to improve fish consumption during the complementary feeding period. To address access related barriers, interventions should focus on promoting sustainable fish production that increases the income of fishers and fishing households. One way of doing this is to encourage use of fishing gear designed to maximize catch of larger mature fish, thereby increasing fisher income and lessening the impact of harmful fishing practices on the marine environment. These practices increase the supply of high quality fish while simultaneously improving the long-term sustainability of fisheries [56]. Another approach to improving access is through outreach efforts to encourage fishermen to bring home fish for family, and especially young child, consumption instead of selling the entire catch. Fishermen could also reserve seafood with lower market value but good nutritional value for household consumption and sharing. Supporting the growth and expansion of *mama karanga* businesses could increase the availability of fish closer to villages. Additionally, bolstering local savings programs and other social protection schemes have been shown to improve food security and boost household income, mitigating the high costs of fish, especially during seasons when supply is low and cost is high [57]. Promoting consumption of other locally available animal sourced foods or preserved forms of fish would reduce the burden of purchasing fish during the off-season while meeting dietary requirements. Finally, attention should be paid to balancing the growth of tourism with the needs of the local population in order to mitigate impacts on fish prices and fisheries exploitation [58].

Our findings point to the need for positive and accurate messaging around the importance of fish during the complementary feeding period. A recent evaluation of the Baby Friendly Community Initiative (BFCI) in Kenya found that the involvement of CHWs in nutrition education programming was a key factor in improving maternal knowledge of feeding practices and overall child health [59]. Success was also attributed in part to cooking demonstrations utilizing local recipes to teach mothers how to prepare and modify animal source foods for young children [60]. A similar approach could be used to train CHWs to promote feeding fish to young children daily as a positive and healthy practice in communities. Cooking demonstrations could be deployed to introduce recipes and methods of fish preparation to reduce the risk of choking on bones and enhance palatability. In line with recommendations from other child feeding studies [61, 62], incorporating community elders and influential family members in nutrition education and messaging could also provide additional support to mothers who rely on them for infant and young child rearing. Such inclusion might go some way toward harmonizing messaging from different sources or at least limiting contradictory messages.

More research is needed to clarify the comparative weights of factors included in the conceptual framework in terms of their influence on the decision to feed young children fish during the complementary feeding period. Additionally, more research on the intergenerational dynamics between caregivers and older community members, as well as intra-household patterns shaping the decision-making process would help to design inclusive interventions that consider the multiple influences on feeding practices.

Overall, this study points to the value of using qualitative methods to understand barriers to consuming highly nutritious foods, like fish, during the complementary feeding period in low income coastal communities. Future research could benefit from the use of these and other participatory approaches to design and test nutrition interventions based on robust community input and feedback. For example, Trials for Improved Practice (TIPS) is an approach that utilizes dialogue-heavy formative research to create and test context-specific and culturally appropriate public health interventions [63, 64]. Ultimately, leveraging community perspectives and input to examine barriers to consuming nutritious foods in coastal Kenya provides necessary context for designing solutions that address key inflection points, thereby improving the chances for improved nutritional outcomes and sustainable change.

## Supporting information

S1 Appendix Maternal and child consumption of foods of fish origin key informant instrument: Caregivers. (DOCX)

S2 Appendix Maternal and child consumption of foods of fish origin key informant instrument: Health workers. (DOCX)
