# Supplementary material for: Fish and complementary feeding practices for young children: Qualitative research findings from coastal Kenya
Source: PLoS One. 2022 Mar 14;17(3):e0265310. doi: 10.1371/journal.pone.0265310 (PMC8920237; doi:10.1371/journal.pone.0265310)
Supplement: S1 Appendix — (DOCX) [file pone.0265310.s001.docx]

Good day (good evening). First, we want to thank you for being here with us. As you know, we are a study on the subject of fish and nutrition of young children. To better understand this topic, we want to know what your opinions are about it.

I want to inform you how we are going to carry out this conversation. First, the conversation will last between an hour and an hour and a half. Second, I'm going to ask you some questions, and we want you to give us your opinions and ideas. There are no right or wrong answers; Any idea or opinion is valid and welcome.

Before starting, it would be good to introduce ourselves and at the same time mention something about the family. I'll start; My name is _____________ and in my family _____________.

1. General – CF & family fish consumption

- How old is your child?
- When did you first give your child solid foods?
- What were the first foods given from 6-12 months?
- What does the child eat now [probe for variety and range of foods given in a week]?
- What foods would you include in a healthy diet for your young child?
- How do you use fish as a food for your family? For your young children? [probe for perceptions of fish in relation to child growth and health]
- What are some positive and negative qualities about fish?
- What types of fish does your family consume most often? Would you be willing to try new kinds of sea foods such as seaweeds, oysters, crabs, etc. (that might protect the environment and help with fish supply in the ocean)?
- In a week, what other “protein” foods does your family consume? [probe for how fish compares in terms of percentage consumed compared to other ASF – eggs, milk, meat]

1. Child fish feeding practices & portion sizes

- How do you prepare the fish for your child?
- Have you heard anyone saying that fish should not be given to young children? What was the reason mentioned?
- How does fish compare to other foods: nourishment (beef, beans, etc.); digestibility, taste [probe do babies like it better than bread?]; ease of preparation; availability; and cost?
- Have you or your child tried other seafoods besides fish [probe for seaweed, shellfish]? Why or why not? If not, would you be willing to try other seafoods? Why or why not

1. Fish access & cost

- Where do you get the fish for your family to eat?
- How much does fish cost from different markets and sources? [probe for differences in individual vs. larger quantities]
- What other animal source foods do you buy for your family – eggs, milk, meat? How does your spending on fish compare with other ASFs?

1. Social Marketing

- How do you learn about healthy foods for your children?
- What would be the best ways for you to learn about healthy foods and preparing them?– mobile phone messages or videos, photo books, talks in health clinics, peer sharing groups, etc.
- Who do you ask for advice about child rearing and child feeding?”: family member, neighbor, health worker, doctor?
- If you were told that fish is healthy and nutritious for children, what reasons can you think of not to follow that advice? (barriers)

1. Intervention ideas – merry-go-rounds & peer support

- Do you currently participate in a merry-go-round savings club [us local term for savings club]? If yes, how is the group organized; how many participate and what are the rules for participation?
- Do you use a mobile phone app for the merry-go-round and/or banking? Mpesa, Airtel, or another mobile service? Why or why not?
- How do you use the money from participation in the merry-go-round?
- Do you participate in a womens/mothers clubs [use local term]? If yes, how often do you meet and what are the topics of conversation (if you wish to share!)? How large is the club and how often do you meet?

1. Child growth and development

- How do you know if a child is growing well? (“ukuaji ya mtoto”?)
- How do you know if a child is developing well? (“maendeleo ya mtoto” ?)
  - What are the markers or signs of development?(“dalili”)
- How do you think that nutrition can help the child grow?
- How do you think that nutrition helps the brain and the child develop?
