# Supplementary material for: Fish and complementary feeding practices for young children: Qualitative research findings from coastal Kenya
Source: PLoS One. 2022 Mar 14;17(3):e0265310. doi: 10.1371/journal.pone.0265310 (PMC8920237; doi:10.1371/journal.pone.0265310)
Supplement: S2 Appendix — (DOCX) [file pone.0265310.s002.docx]

Good day (good evening). First, we want to thank you for being here with us. As you know, we are a study on the subject of fish and nutrition of young children. To better understand this topic, we want to know what your opinions are about it, especially in the context of your role as a health worker.

I want to inform you how we are going to carry out this conversation. First, the conversation will last between an hour and an hour and a half. Second, I'm going to ask you some questions, and we want you to give us your opinions and ideas. There are no right or wrong answers; Any idea or opinion is valid and welcome.

Before starting, it would be good to introduce ourselves and at the same time mention something about the family. I'll start; My name is _____________ and in my family _____________.

1. General

- What is your role in the health care system?
- How do you interact with pregnant/lactating women and mothers of young children? [probe for frequency of interaction, place this occurs, and forms of communication]
- How is nutrition part of your practice in healthcare? What problems of malnutrition do you see, and what are the responses?
- What other health problems do you see most often in pregnant/lactating and young children experience?
- What are some positive and negative qualities about fish nutrition?

1. Messages around fish feeding practices

- Do you promote fish as a food for pregnant/lactating women and young children? If so, why and how? [probe for perceptions of fish in relation to child growth and health]
- What message are communicated to with regards to fish preparation practices or dishes that include fish?
- How do you discuss hygiene and sanitation practices?
- Do you think mothers are aware of health concerns about spoiled fish? What do they do to prevent this from happening in their household?
- Have you heard anyone saying that fish should not be given to young children? What was the reason mentioned?
- How does fish compare to other foods: nourishment (beef, beans, maize, etc.); digestibility, taste; ease of preparation; availability; and cost?
- From your perspective, what are some of barriers and opportunities for mothers to fish nutrition?

1. Social Marketing

- What would be the best ways for you to promote dietary diversity and healthy foods?– mobile phone messages or videos, photo books, talks in health clinics, peer sharing groups, etc.
- What other “protein” foods are promoted in your practice? [probe for how much fish factors into this food group? Is it included as an animal source food with eggs, milk, meat?]
- Where do your mothers seek advice about child rearing and feeding? [probe for family members, neighbors, health workers, doctor, etc.]
- Would you be willing to promote new kinds of sea foods such as seaweeds, oysters, crabs, etc. (that might protect the environment and help with fish supply in the ocean)?
